# Interleukin-16 Gene Polymorphisms Are Considerable Host Genetic Factors for Patients' Susceptibility to Chronic Hepatitis B Infection

**DOI:** 10.1155/2014/790753

**Published:** 2014-11-26

**Authors:** Sara Romani, Seyed Masoud Hosseini, Seyed Reza Mohebbi, Shabnam Kazemian, Shaghayegh Derakhshani, Mahsa Khanyaghma, Pedram Azimzadeh, Afsaneh Sharifian, Mohammad Reza Zali

**Affiliations:** ^1^Gastroenterology and Liver Diseases Research Center, Shahid Beheshti University of Medical Sciences, Tehran, Iran; ^2^Department of Microbiology, Faculty of Biological Sciences, Shahid Beheshti University, Tehran, Iran; ^3^Basic and Molecular Epidemiology of Gastroenterology Disorders Research Center, Shahid Beheshti University of Medical Sciences, Tehran, Iran

## Abstract

Host genetic background is known as an important factor in patients' susceptibility to infectious diseases such as viral hepatitis. The aim of this study was to determine the effect of genetic polymorphisms of interleukin-16 (IL-16) cytokine on susceptibility of hepatitis B virus (HBV) infected patients to develop chronic HBV infection. Genotyping was conducted using PCR followed by enzymatic digestion and RFLP (restriction fragment length polymorphism) analysis. We genotyped three single nucleotide polymorphisms (SNPs) in the *Il-16* gene (rs11556218 T>G, rs4778889 T>C, and rs4072111 C>T) to test for relationship between variation at these loci and patients' susceptibility to chronic HBV infection. Allele frequency of *Il-16* gene rs4072111 and rs11556218 was significantly different between chronic HBV patients and healthy blood donors. Genotype frequency of rs4778889 polymorphism of *Il-16* gene was significantly different when chronic HBV patients and HBV clearance subjects were compared. Our results showed that *Il-16* gene polymorphisms are considerable host genetic factors when we chase biomarkers for prognosis of HBV infected patients.

## 1. Background and Objectives

More than 350 million individuals around the world are infected with hepatitis B virus. The diagnosis of chronic infection is made by a combination of serology, viral, and biochemical markers [1]. Prevalence of anti-HBc and HBsAg in the United States at 2006 is estimated to be 4.7% and 0.27%, respectively [2].

Iran is located on intermediate endemic region for hepatitis B infection and prevalence of HBsAg positivity among general adult population in about 3 to 10 percent in different regions [3]. World Health Organization (WHO) and Center for Disease Control and Prevention (CDC) reported that prevalence of chronic hepatitis B infection in Iran ranges between 2 and 7 percent [4].

Occult HBV infection is reported among 7 to 13 percent of anti-HBc positive and/or anti-HBs positive subjects, but this type of viral hepatitis is seen in 0 to 17 percent of healthy blood donors. When we investigate the susceptibility factors for HBV clearance this phenomenon could have confounder effect. So HBV-DNA PCR test must be performed for all study subjects to obviate this issue [5].

Interleukin-16 (*Il-16*) is a multifunctional cytokine with the role of immune response synchronization and direction [6]. The mature secreted form of this protein acts as a ligand for CD4^+^ cells and this ligand-receptor binding leads to activation of a key intracellular pathway that regulates T cell proliferation [7]. The major* Il-16* related functional proteins are STAT6, interleukin-4 (*Il-4*), and tumor necrosis factor-*α* (TNF-*α*) [8].

Some current efforts to prevent the populations from the infectious diseases such as viral hepatitis have focused on finding powerful genetic susceptibility biomarkers [9]. In recent years few polymorphism markers have been found in association with viral hepatitis. SNPs located in* HLA-DPA1* and* HLA-DPB1* genes were identified as protective factors for chronic hepatitis B infection [10] and SNPs of* IL28B* gene were significantly related to spontaneous clearance of hepatitis C infection [11].

HBV clearance is in connection with powerful CD4^+^ T cells and* Il-16* plays a critical role in T cell regulation [12]. Due to this, phenomenon variations occurring in* Il-16* gene sequence could affect its function and cause deregulation in immune response against viral hepatitis [13].

Present study was designed to investigate the relationship between three SNPs of* Il-16* gene and patients' susceptibility to chronic hepatitis B infection and to determine the effect of* Il-16* gene polymorphisms on development of chronic HBV infection.

## 2. Methods

### 2.1. Study Population

Seven hundred and forty-four individuals were enrolled in the genotyping procedure including 245 chronic HBV patients, 105 HBV clearance subjects, and 394 healthy controls. Inclusion criteria for chronic hepatitis B patients group were HBsAg positive test for at least 6 months and HBV infection was confirmed by detection of serum HBV DNA using PCR method. The patients with these characteristics were excluded from study: HCV or HIV coinfection and history of autoimmune diseases. HBV clearance group consisted of individuals with anti-HBc positive and HBs-antigen negative tests. All healthy control subjects were anti-HBc, HBs-antigen, and HBV-DNA PCR negative and have not met these criteria, any history of hepatitis or liver dysfunction, autoimmune diseases, and HCV or HIV.

### 2.2. Single Nucleotide Polymorphisms

According to literature review and basic information about the* Il-16* cytokine, we selected three SNPs in the* Il-16* gene sequence including rs11556218 T>G, rs4778889 T>C, and rs4072111 C>T. The minor allele frequency (MAF) of all three selected SNPs was greater than 0.05 according to previous studies.

### 2.3. Genomic DNA Purification and Genotyping

Blood samples were collected from all study subjects in EDTA coated tubes. Genomic DNA was extracted from whole blood using standard phenol chloroform method as previously described by Sambrook [14]. Genotype determination for three selected SNPs was performed by PCR-RFLP method as previously described by Gao et al. [15] Primer sequences and RFLP material are presented in Table 1. In brief pure PCR products were obtained using specific primer pairs (Bioneer, South Korea) for each SNP; and enzymatic digestion for each one was performed using specific restriction endonucleases (Fermentas, Latvia). Digested DNA was analyzed on 3% low electroendosmosis agarose gel (Agarose LE, Roche, Germany).

### 2.4. Direct Sequencing

To confirm the results of RFLP analysis we performed direct sequencing using chain termination method and 3130*xl* genetic analyzer instrument (Applied Biosystems, USA) for 5% of our samples as duplicate genotyping.

### 2.5. Statistical Analysis

Statistical analyses were carried out using IBM SPSS software version 20 (IBM SPSS Statistics 20; SPSS, Chicago, IL). *P* values less than 0.05 were considered as significant. To compare three included groups (HBV cases, HBV clearance, and healthy controls) for their genotype and allele status we performed Chi square test and to consider the simultaneous effect of genotype and allele along with gender and age (as probable confounder variables) we conducted a logistic regression test. To compare the mean age between three studied groups we used one-way ANNOVA.

## 3. Results

Among 245 chronic HBV patients 157 (64.1%) individuals were male and 88 (35.9%) were female and from 394 healthy controls 205 (52%) were male and 189 (48%) were female; so our third group (HBV clearance) consisted of 64 male and 41 female, total 105 subjects. Mean age in chronic HBV group was 49.15 ± 15.50 years, in healthy control group was 45.26 ± 16.42 years, and in HBV clearance group was 42.2 ± 14.96 years; there was a significant difference between three groups according to age and gender status (*P* value < 0.05). In order to control the probable confounding effects of age and gender we performed data adjustment using logistic regression.

All subjects were genotyped for three SNPs of* Il-16* gene sequence. When comparing chronic HBV patients with healthy blood donors, we found a significant association between T allele of rs4072111 polymorphism and higher risk of chronic disease development (*P* value: 0.029, OR: 1.471, and 95% CI: 1.039–2.081). Frequency of TG and GG genotypes and G allele distribution in rs11556218 polymorphism were also significantly different between two study groups, but our results showed no statistically significant difference between allele and genotype frequency of rs4778889 polymorphism between chronic HBV patients and healthy control group. These data are summarized in Table 2. Despite these findings, we found a statistically significant relationship between the rs4778889 CC genotype (rare genotype) and three times higher risk for chronic HBV infection, when we compare chronic HBV and HBV clearance groups (*P* value: 0.035, OR: 3.723, and 95% CI: 1.100–12.602). Summary of genotyping data of chronic HBV and HBV clearance groups is shown in Table 3.

## 4. Discussion

The mechanisms involved in the patients' susceptibility to chronic hepatitis B infection are not well understood. Clearance or pathogenesis of HBV infection is expected to be multifactorial affected by environment, viral factors, and host genetic variations. The products of many human genes and their downstream effectors influence host defense against HBV infection and person to person differences in susceptibility to chronic hepatitis disease.

Precursor of human* Il-16* protein contains 631 amino acids and is constitutively synthesized in unstimulated T cells. This peptide goes through proteolytic processing that results in a 121-amino acid bioactive molecule [5]. Almost all 121 amino acids of this peptide are involved in PDZ domains and this phenomenon makes it a unique structure [16].

*Il-16* is secreted by several types of cells and its association with recruitment of CD4^+^ immune cells to location of inflammation. Involvement of* Il-16* in pathogenesis of viral hepatitis is in connection with its receptor (CD4) [17].* Il-16* is produced by a variety of immune cells in addition to CD4^+^ and CD8^+^ T cells [18]. Biological activities of* Il-16* cytokine are chemotaxis of CD4^+^ cells, upregulation of CD25 protein, and secretion of interleukin-1b (*Il-1b*), interleukin-4 (*Il-4*), and tumor necrosis factor-*α* (*TNF-α*). On the other hand, activation of STAT6 protein is a common function in CD4^+^ cells, and STAT6 together with* Il-4* are involved in T cell mediated hepatitis. This pathway of events is consistent with the fact that immune responses related to T lymphocytes are important in viral hepatitis [19, 20].

There is limited data about the association of SNPs in the* Il-16* gene sequence and risk of chronic hepatitis B infection, whereas the role of cellular immunity in dealing with hepatitis HBV infection is clear and on the other hand CD4 (receptor of* Il-16*) is involved in this process [21, 22]. So it might be considered that* Il-16* plays a role in immune response against HBV infection.

According to the results of our study, rs11556218 T>G, rs4778889 T>C, and rs4072111 C>T polymorphisms of* Il-16* gene are associated with patients' susceptibility to chronic hepatitis B infection. As we showed in the results section rs4072111 and rs11556218 polymorphisms are in relationship with higher susceptibility of general population for development of chronic HBV and rs4778889 polymorphism which is associated with higher risk of chronic HBV among HBV infected individuals.

A recent published study on Chinese patients suffering from hepatitis C virus (HCV) disease revealed that there is an association between STAT6 SNPs and patients' susceptibility to sustained viral response. These outcomes suggest that STAT6 SNPs are prospective genetic biomarkers for HCV prognosis [23].

Li et al. reported that the genetic variations (SNPs) of* Il-16* are significantly related to chronic HBV related hepatocellular carcinoma (HCC). These results also showed that rs11556218 TG and GG genotypes of the* Il-16* gene contributed in patients' susceptibility to chronic hepatitis B when using healthy subjects as controls [24].

In our previous study we have shown that there is a significant relationship between the micro-RNA binding site polymorphism of the* Il-16* gene and risk of colorectal cancer (CRC) [25]. Our previous study also suggested that rs11556218 T>G and rs4778889 T>C polymorphisms have influence on the altered risk of CRC [26].

In conclusion, HBV infected patients with anti-HBc Ab positive test who have had CC genotype rs4778889 are more susceptible to establish a chronic disease. We can also conclude that rs4072111 and rs11556218 polymorphisms are suitable susceptibility biomarkers for development of chronic HBV infection. Our results are in concordance with previous studies and we suggest that* Il-16* is related to hepatitis B infection and* Il-16* gene polymorphisms are considerable host genetic factors for patients' susceptibility to chronic hepatitis B infection.

## Figures and Tables

**Table 1:** PCR and RFLP information for the SNPs of interleukin-16 gene.

Polymorphism	Primer	PCR (bp)	Restriction enzyme (incubation temp °C)	Restricted fragments' size (bp)
rs4072111 C/T	F: 5′-CACTGTGATCCCGGTCCAGTC-3′ R: 5′-TTCAGGTACAAACCCAGCCAG C-3′	164	BsmAI (55)	C: 164 T: 140 + 24

rs11556218 T/G	F: 5′-GCTCAGGTTCACAGAGTGTTTCCATA-3′ R: 5′-TGTGACAATCACAGCTTGCCTG-3′	171	Nde I (37)	T: 171 G: 147 + 24

rs4778889 T/C	F: 5′-CTCCACACTCAAAGCCTTTTGTTCCTAT${\underline{\text{G}}}^{\text{a}}$ A-3′ R: 5′-CCATGTCAAAACGGTAGCCTCAAGC-3′	280	AhdI (37)	T: 280 C: 246 + 34

^a^The underlined base in the forward primer is different from that of the original sequence and serves as the introduction of a recognition site for the restriction enzyme AhdI.

**Table 2:** Allele and genotype frequency of three SNPs among chronic HBV patients versus healthy control subjects.

SNP	Variable	Healthy control (*n* = 394) *n* (%)	HBV patient (*n* = 245) *n* (%)	Adjusted^*^ OR (95% CI), *P*_value_
rs4072111	Genotypes			
	CC	316 (80.2)	181 (73.9)	1.00 (reference)
	CT	74 (18.8)	58 (23.7)	1.391 (0.937–2.066), 0.102
	TT	4 (1.0)	6 (2.4)	2.929 (0.787–10.894), 0.109
	Alleles			
	C	706 (89.6)	420 (85.7)	1.00 (reference)
	T	82 (10.4)	70 (14.3)	**1.471** (**1.039–2.081**), **0.029**

rs11556218	Genotypes			
	TT	124 (31.5)	43 (17.6)	1.00 (reference)
	TG	215 (54.6)	147 (60)	**2.028 ** (**1.344–3.061**), **0.001**
	GG	55 (14)	55 (22.4)	**2.894** (**1.722–4.864**), **0.000**
	Alleles			
	T	463 (58.8)	233 (47.6)	1.00 (reference)
	G	325 (41.2)	257 (52.4)	**1.574** (**1.250–1.982**), **0.000**

rs4778889	Genotypes			
	TT	264 (67)	156 (63.7)	1.00 (reference)
	TC	111 (28.2)	84 (34.3)	1.303 (0.916–1.853), 0.140
	CC	19 (4.8)	5 (2)	0.439 (0.159–1.216), 0.113
	Alleles			
	T	639 (81.1)	396 (80.8)	1.00 (reference)
	C	149 (18.9)	94 (19.2)	1.027 (0.767–1.375), 0.860

^*^Adjusted for confounder variables: age and gender.

**Table 3:** Allele and genotype frequency of three SNPs among chronic HBV patients versus HBV clearance subjects.

SNP	Variable	HBV clearance (*n* = 105) *n* (%)	HBV patient (*n* = 245) *n* (%)	Adjusted^*^ OR (95% CI), *P*_value_
rs4072111	Genotypes			
	CC	86 (81.9)	181 (73.9)	1.00 (reference)
	CT	17 (16.2)	58 (23.7)	0.577 (0.307–1.082), 0.086
	TT	2 (1.9)	6 (2.4)	0.814 (0.142–4.672), 0.817
	Alleles			
	C	189 (89.6)	420 (85.7)	1.00 (reference)
	T	21 (10.4)	70 (14.3)	0.651 (0.378–1.121), 0.122

rs11556218	Genotypes			
	TT	16 (15.2)	43 (17.6)	1.00 (reference)
	TG	63 (60)	147 (60)	1.118 (0.567–2.203), 0.748
	GG	26 (24.8)	55 (22.4)	1.298 (0.594–2.836), 0.513
	Alleles			
	T	95 (45.2)	233 (47.6)	1.00 (reference)
	G	115 (54.8)	257 (52.4)	1.109 (0.788–1.561), 0.509

rs4778889	Genotypes			
	TT	68 (64.8)	156 (63.7)	1.00 (reference)
	TC	29 (27.6)	84 (34.3)	0.785 (0.459–1.342), 0.376
	CC	8 (7.6)	5 (2)	**3.723 ** (**1.100–12.602**), **0.035**
	Alleles			
	T	165 (78.6)	396 (80.8)	1.00 (reference)
	C	45 (21.4)	94 (19.2)	1.144 (0.751–1.744), 0.531

^*^Adjusted for confounder variables: age and gender.
